# Effect of mobile-based self-management application on stroke outcomes: a study protocol for triple blinded randomized controlled trial

**DOI:** 10.1186/s12911-022-02033-y

**Published:** 2022-11-11

**Authors:** Hamidreza Tadayon, Mehrdad Farzandipour, Ehsan Nabovati, Hossein Akbari, Seyed Ali Masoud

**Affiliations:** 1 Health Information Management Research Center, Kashan University of Medical Sciences, Kashan, Iran; 2 Department of Health Information Management & Technology, Kashan University of Medical Sciences, Kashan, Iran; 3 Department of Biostatistics, Kashan University of Medical Sciences, Kashan, Iran; 4 Department of Neurology, Kashan University of Medical Sciences, 5th Km Qotb-e Ravandi Blvd, Kashan, Iran

**Keywords:** Mobile applications, Self-care, Stroke, Telemedicine, Telerehabilitation

## Abstract

**Background:**

Stroke is the main leading cause of long-term disabilities in the world. This protocol will be implemented for a study to evaluate the effects of an Android-based self-care application on patients with stroke.

**Methods:**

The first stage will include the development of an android-based application using JAVA programming language for developing the user interface and ASP.NET Core for developing Web server. The second stage will be conducted using triple blinded randomized clinical trial (RCT). The sample size will include 60 patients with recent stroke and partial paralysis of limbs, who will be divided into two groups of intervention and control through permuted block randomization method. Patients in both groups will receive usual medical care, but those in the intervention group will also use an Android-based application for a period of two months. Outcomes will be assessed using valid and reliable questionnaires.

**Discussion:**

The assessed outcomes will include stroke severity using National Institute of Health Stroke Scale (NIHSS) score, ability to perform activities of daily living using Barthel Index (BI) score, depression rate using Beck Depression Inventory (BDI-II) score, quality of life using EQ-5D-3L score, medication adherence using Modified Morisky Medication Adherence Scale (MMAS-8) score, patient satisfaction using Patient Satisfaction Questionnaire (PSQ) score and the number and type of complications in patients in two groups. These outcomes will be assessed at baseline, after two months and after three months from the beginning of the intervention. Intervention effects on the measured variables will also be evaluated using appropriate statistical tests based on the type of variable distribution. Potential consequences of the study might be the improvement of the measured variables in the intervention group compared to that of the control group. The expected results are that the intervention may significantly improve the status of the measured variables in the intervention group compared to that of the control group. If the outcomes of the intervention group do not change significantly compared to those of the control group, it can be due to different reasons. However, this can most likely be attributed to incorrect or insufficient use of the application by patients.

*Trial registration*: This protocol is registered in the Iranian registration of clinical trial (IRCT) on November 7, 2020 with the code IRCT20201015049037N1. URL: https://irct.ir/trial/51674

## Background

The incidence of stroke in 2017 (based on findings from 21 regions of the world) was 11.9 million cases, and the disease is the second leading cause of death worldwide and the main leading cause of long-term disabilities [1]. According to the World Health Organization (WHO), of the 56 million deaths that occur worldwide each year, 10.8% are due to stroke [2]. Stroke includes a group of pathological disorders of cerebral arteries characterized by decreased neurological and non-convulsive function due to cerebral ischemia or intracranial hemorrhage [3]. Stroke has a variety of consequences for patients [4] and stroke survivors usually experience a wide range of symptoms related to motor function, speech, swallowing, vision, sensation and perception, and their recovery can be slow and incomplete [5, 6]. These symptoms often lead to limited participation in home and community activities. Rehabilitation procedures for these people are often long-term and require a lot of resources. However, only half of survivors have access to rehabilitation services after discharge [7]. In a qualitative study, Anderson et al. [8] concluded that mobile-based applications could improve self-care in patients with chronic conditions. Other studies have shown the impacts of mobile-based training on improving stroke outcomes [9, 10]. An increasing number of Randomized Clinical Trials (RCTs) have evaluated the effectiveness of telerehabilitation interventions. Nevertheless, it is difficult to draw conclusions about the effects of these interventions since the type of interventions and comparisons performed vary greatly from study to study. Some studies have shown the effects of mobile-based applications on improving and enhancing some of the measured outcomes in patients after stroke [11–14] while other studies showed the use of these applications does not make a significant difference compared to usual care. [15, 16]. In addition, the results of a number of studies do not seem to be generalizable and might be at the risk of being biased, and accordingly more studies are needed to yield more definitive conclusions [7]. Therefore, in this study, we intend to first develop a mobile-based application and then solve its usability problems by expert-based usability evaluation. Finally, an interventional study will evaluate the effects of this application on patients with stroke. The effect assessment of the application will be done by focusing on the three main parts of the International Classification of Functioning, Disability and Health (ICF), namely the physical structure, individuals’ activities and social participation. To write this paper, the conventional SPIRIT (Standard Protocol Items: Recommendations for Interventional Trials) checklist and guidance on reporting e-health and m-health trials written by the CONSORT-EHEALTH (Consolidated Standards of Reporting Trials of Electronic and Mobile Health Applications and online Telehealth) group [17] were used.

## Methods

This RCT will be done in two stages. The first stage will include the development of an android-based application, with the second stage being the evaluation of the effects of this application on patients with stroke.

### Stage 1: application development

**Fig. 1:**
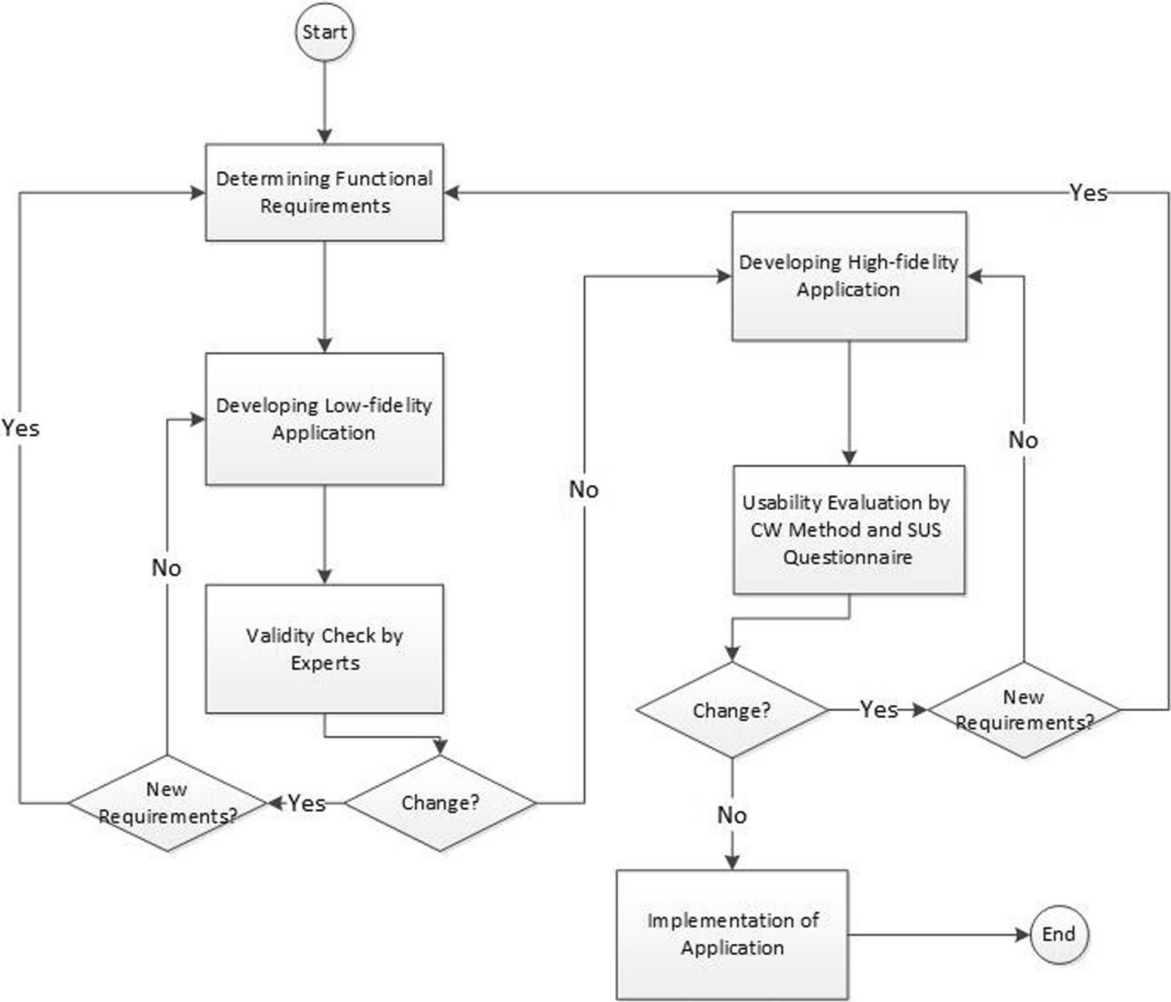
Method of software development

Application development will be based on the model presented by Chavez et al. in 2019 [18] (Fig. 1). This model is a combination of iterative and incremental models in system development. According to this model, software development will be done in three different phases, which include the following:

- Determining functional requirements
- Designing a low-fidelity prototype and evaluating its validity
- Designing a high-fidelity prototype and evaluating its usability

#### Determining functional requirements

In this phase, the functional requirements of the application will be determined. Functional requirements will be obtained through the following four methods:

- Selecting clinical guidelines
- Reviewing similar studies and applications
- Needs assessment of patients
- Needs assessment and localization of requirements based on expert opinion

In this phase, based on the existing stroke self-care guidelines, one of the clinical guidelines approved by three neurologists will be selected at first and the functional requirements of this guideline will be extracted by researchers. Other functional requirements will also be extracted using a review study on applications done by researchers and similar studies. In this regard, some systematic review studies have been conducted [7, 19–22] which will also be reviewed by researchers. Needs assessment of patients will be done as the most important stakeholder group of the application. For this purpose, a semi-structured interview will be used with patients who have previously suffered from stroke and partial paralysis and have now partially recovered. Due to the specific conditions of the patients, the interview will be conducted in a maximum of 20 min, but the number of sessions can be increased to two or more sessions in order to assess patients’ needs. Sample interview questions are stated in Table 1. These patients are required to have at least a diploma and be able to work with a smartphone. Patient and caregiver interviews will continue until data saturation is reached and no new data should be produced in the interview. Samples will be selected by convenience sampling method. Finally, all these requirements will be prepared in the format of Lawshe approach [23] and will be given to the experts. At this stage, a questionnaire containing functional requirements and information content of the application will be given to neurologists, occupational therapists and physiotherapists for the purpose of localization. Delphi technique [24] will be used in this step. Moreover, for needs analysis, 15–21 neurologists, physiotherapists and occupational therapists who are faculty members of the country's medical universities and have at least three years of professional experience will be consulted. At this stage, for each requirement, the necessity, appropriateness, clarity and relevance will be evaluated and in terms of necessity (CVR: Content Validity Ratio), three levels (essential and useful, useful but unnecessary, non-useful) and in terms of appropriateness, clarity and relevance (CVI: Content Validity Index) four levels (from very high with a score of four to very low with a score of one) will be defined. Questions with a CVR score of higher than the base number (0.62 for ten individuals) based on Lawshe table will be approved and those with a CVR score of less than that will be removed. According to CVI, questions that have scores of higher than 0.79 will be approved and those with scores between 0.7 and 0.79 will be re-reviewed, corrected and returned to the experts and finally those with scores less than 0.7 will be rejected. This step will be repeated until the final validity of the questionnaire content is confirmed. The approved questionnaire will be provided to the experts in the next step and the experts' answers to the components of the questionnaire will be scored from 1 to 5 (very low = 1, low = 2, medium = 3, high = 4 and very high = 5) and then the average score of each item will be calculated. Minimum score for accepting each component will be considered as 3.75. If the score obtained for each component is between 2.5 to 3.74, that component will be modified and reviewed again. Components with a score of less than 2.5 will also be removed from the application content.

**Table 1:** Example of questions for patient interviews

No	Question
Q1	During your illness, did you have any problems in doing activities of daily living (such as eating, going to the toilet, dressing, etc.)? Explain your problems
Q2	Did you suffer from complications caused by the disease such as bedsores, urinary and fecal incontinence, shoulder pain, falls, depression and other complications? Explain them and explain how you managed to improve them
Q3	Were you prescribed physiotherapy sessions? What exercises did you do during the physiotherapy treatment?
Q4	Did you have problems walking and maintaining your balance? Explain your problems
Q5	During your illness, did you feel the need to communicate with your doctor by phone? For what cases did you feel this need?
Q6	During your illness, did you feel the need for training to do some work? What training was needed more?
Q7	Did you need special training regarding prescribed drugs? (Knowing the side effects of drugs, changing the dosage of drugs, the duration of continuing to take the drug, or stopping the use of drugs, changing the type of drugs). Explain

The interview questions will not be limited to these questions and the interview will continue in depth until the patient's needs are acquired

#### Development of low-fidelity interface

In this phase, based on the feedback and opinions received from experts in the previous step, first the items and how the application works as a conceptual model will be designed and then the low-fidelity user interface (how to display the content of the application and the relationship between humans and smartphones) on android platform will be developed using the appropriate programming language. Then, a primary prototype will be created and its validity will be evaluated. After implementing functional requirements on the prototype for checking validity, six experts will review the application and evaluate the extent to which functional requirements and goals are met by this prototype. These experts will include two neurologists, two physiotherapists and two occupational therapists with at least three years of specialized work experience in the field of treatment and rehabilitation of patients with stroke.

#### Development of high-fidelity interface

At this phase, based on the final version of the prototype obtained from the previous phase, the high-fidelity prototype will be developed by JAVA programming language. A web server will also be developed using ASP.NET Core. In order to evaluate the usability of the prototype, Cognitive Walkthrough (CW) method will be used based on the method proposed by Polson and Lewis [25]. By identifying and introducing tasks and actions and expressing them step by step, this method will enable those evaluators who have limited background knowledge about the system to identify usability problems properly [26]. For this purpose, a certain number of scenarios that represent the most important and frequent application functions will be identified in harmony with the opinion of three neurologists. For each scenario, the main objective, sub-objectives, sequence of steps to do the tasks and system responses will be prepared and approved by the researchers. Then, the program will be provided to the evaluators. Five individuals will be chosen to do the evaluation through non-probability quota sampling. Evaluators are required to have at least a master's degree in health information technology/management or medical informatics and have 2 years of professional experience in evaluating health information systems. They will independently perform the sequence of steps for each scenario through the program's user interface. Each evaluator will put themselves in the position of the patient and observe the program from the patient's point of view; in case of any problems in performing the task steps, it will be expressed by evaluators. This will happen at the presence of the researcher as an observer who will write the following items in data collection forms: evaluator's comments, questions and uncertainties, an explanation of the usability problem identified by the evaluator, location of the problem in the system and the time to perform each scenario. At the end of each evaluation process, each evaluator will review the list of the related problems and suggest any solutions to solve potential issues. Then, in a meeting, separate lists of evaluators will be compared and all of the identified problems will be put on the list of main problems. Next, any duplicate problems will be eliminated and the evaluators will be asked to assess the severity of the problems independently. This rating will be based on three criteria including frequency of the problem, impact of the problem and continuity of the problem. Scoring for each problem and criterion will be done in 5 degrees ranging from 0 (no problem) to 4 (severe problem). Then, the average of the identified problems will be calculated and relevant problems will be listed in order of intensity followed by feedback given to application designers. Also, in order to confirm usability of the application by users, the System Usability Scale (SUS) questionnaire will be used and will be distributed among 30 users who have previously had a stroke and have been working with the application for a week. This questionnaire will include 10 items (SUS01 to SUS10) with 5-point Likert scale and SUS score of 70 and above will be considered acceptable [27]. SUS scores will be calculated as follows:

SUS Score = 2.5 (20 + SUM (SUS01, SUS03, SUS05, SUS07, SUS09) − SUM (SUS02, SUS04, SUS06, SUS08, SUS10)). According to this formula, SUS01 means item number one in the questionnaire, and the list will continue. The validity and reliability of this questionnaire have been confirmed in previous studies and its average Cronbach's alpha coefficient was estimated to be 0.91 [28].

The results of users' comments will also be given to the designers. This step will be repeated until the application is approved by evaluators. The output of this phase will be the final version of the application, which will be ready to be implemented in a live environment. Support services including bug fixation, content changes and other application updates will be presented by system support staff/admin.

### Application testing

At this stage, the unit test of the final application will be performed by researchers and different modules of the application will be tested separately to confirm correct performance, which can help to eliminate any potential defects. In the next step, application integrity test will be performed by researchers. For this purpose, one researcher will put him/herself in the position of the patient and another researcher in the position of the physician. Feedback on probable defects of the application will be given to application designers.

### Implementation and evaluation of application in the live environment (clinical trial)

#### Study design and setting

At this stage, an interventional study will be conducted through patient blinding by an assessor physician and analyst to evaluate the effects of the application on patient self-care. The designed application will be installed on patients’ smartphones and the necessary training to use it will be given to the patients and their caregivers. This application will support Android 5 and above. The study population will include patients referring to one of the general hospitals in Kashan where sampling can be done in a convenient way. Patients with hemiplegia or hemiparesis caused by a recent stroke will be chosen for this purpose. Either the patient or his/her patient caregiver must have an Android-based smartphone and be able to work with it. At least 72 h must have passed since the stroke (The acute phase of the disease must have passed) and patients must have adequate consciousness and hearing and be willing to participate in the study. Exclusion criteria will include:

- Patients who have some levels of consciousness disorder
- Patients with cognitive disorders such as dementia or memory impairment
- Patients with global aphasia

The formula “n = [(Z_1−α/2_ + Z_1−β_)^2^ * (σ_1_^2^ + σ_2_^2^)]/($\overline{x }$_1 _− $\overline{x }$_2_)^2^” with the reliability of 95% and the test power of 90% based on the mean and standard deviation of baseline NIHSS obtained in the same study [29] (Z_1−α/2_ = 1.96, Z_1−β_ = 1.282, σ_1_, σ_2_ = 8, $\overline{x }$_1_ = 5, $\overline{x }$_2_ = 12) was calculated as 27 patients, which increased to 30 patients according to the available facilities which, with a 10% sample dropout, will increase to 33 individuals. Accordingly, two groups each including 33 patients (intervention and control) with stroke (66 patients in total) who meet the inclusion criteria will be selected.

#### Randomization

Randomization will be done by block method (Permuted Block Randomization) in a way that first all foursome blocks which include two codes A and B will be prepared (6 blocks) and then, using a table with random numbers, random blocks will be selected by placement (15 blocks). These blocks will contain up to 60 codes including A and B, each of which will be randomly assigned to either the control or the intervention group. This table will be generated by MS Excel 2019 and function of RANDBETWEEN (1,6).

#### Blinding

For the purpose of blinding, an application will be installed on the smartphones of patients of both intervention and control groups. The program installed for patients in the control group will only contain online questionnaires that patients need to complete and submit at specified intervals. Online questionnaires will include a number of questions used to measure the target variables. However, the program installed on the phones of participants in the intervention group will include all the capabilities and requirements of the self-care program designed in the design phase. Patients will not be familiar with the nature and content of the installed program on the opposite group’s phones. During program installation, someone other than the assessor physician will install the program for patients or their caregivers. Furthermore, during the assessment process, the same person will record the results of the physician evaluation and the scores related to each patient. Therefore, the assessor physician will not know which group a given patient belongs to. Numeric codes for intervention and control groups will also be used for analysis, and the letters A and B will be used for the control and intervention groups when information is sent to the analyst.

#### Intervention

**Fig. 2:**
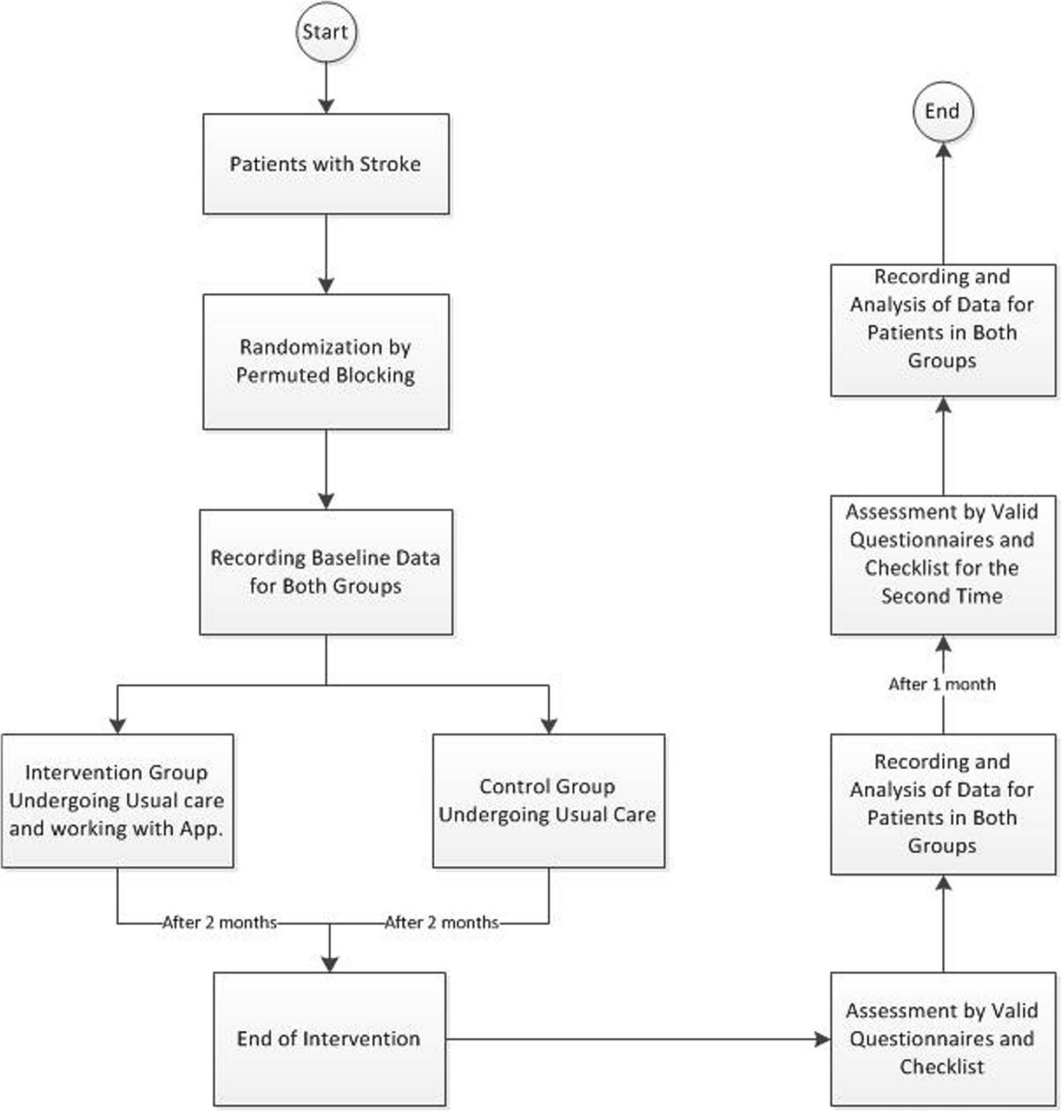
Protocol for interventional trial

Intervention for patients in the intervention group (in addition to usual care) will include an Android-based application that has all the functional requirements approved by the experts in the analysis and software design phases. This application will contain audio, video, textual files and games to improve lower and upper limb function and activities of daily living, prevent and reduce depression, reduce and manage pain and other possible complications of stroke, medication reminders and whatever playing a role in improving patient outcomes, and will be designed according to experts' opinion. Patients will be advised to use the application 5 days a week and for a period of 1 h each day. The duration of the intervention will be 8 weeks (40 sessions in total). A systematic review study [21] showed that the duration of intervention to improve physical activities using mobile phones was between 4 and 6 weeks, which could be extended to 10 weeks for greater effectiveness. According to the conditions in the country and the opinion of experts and follow-up protocols of patients, intervention for more than 8 weeks was not recommended due to the possibility of excessive sample fall. Patients will access the application by creating valid accounts. Account creation by patients and physicians will be done by the system admin and demographic information will be recorded for them; however, the usernames defined for users cannot be changed by them. The application can record the number of times and hours of application use by the user. In order to learn how to use the application, users can contact one of the research team members. A member of the research team will also make weekly phone calls to make sure patients know how to use the application. Intervention for patients in the control group will include usual medical care based on the opinion of the responsible neurologist (This can include physiotherapy or occupational therapy). An application will be installed on the smartphones of these individuals which lacks the main capabilities of the application and only contains electronic questionnaires. Installation of this application will be done only for blinding purposes. Intervention duration and the time of measuring the outcomes will be the same for both groups. Figure 2 shows the flowchart of protocol for interventional trial. Re-stroke will be recorded in both groups and finally it will be considered as one of the complications of stroke in both groups. Nevertheless, an individual with re-stroke during the intervention due to entering the acute phase of the disease will be excluded from the study and replaced by another individual. To replace the new individual, the researcher will return to the hospital and replace another person who meets the study inclusion criteria (In case of excessive sample fall). The research hypothesis is that using the proposed intervention along with usual care can improve stroke outcomes. Therefore, this study can be considered as a superiority trial. Routine rehabilitation interventions (such as electrotherapy) cannot be eliminated at this time. Therefore, no hypothesis can be defined for this intervention alone.

#### Data collection

The required data will be collected using valid and reliable questionnaires that are introduced as follows. Data will be collected in three stages: prior to intervention (before the intervention and at the time of discharge), two months after the intervention and three months after the intervention (in the doctor's office or in the hospital outpatient clinic). Data from National Institute of Health Stroke Scale (NIHSS) and Barthel Index (BI) questionnaires and stroke complication checklist will be filled out by the attending neurologist and other questionnaires will be filled out by patients as self-report. Completion of questionnaires by patients will be done electronically and sent online through the application. Therefore, this survey will be considered as a “closed survey” since only registered users with a valid account can access the questionnaires and submit them. Application features will be inactive until patients, complete questionnaire items. After completion, application features will be activated and the questionnaire will be deactivated to prevent response duplications. The questionnaires will be reactivated two months and three months later. In order to ensure that no data will be missing, all fields and questions will be defined as mandatory in electronic questionnaires and patients will be warned to answer all questions before sending the questionnaire.

All online questionnaires will exactly be designed the same as paper questionnaires in terms of appearance, content and sequence of questions. That is, all the questions will be placed consecutively and not on separate pages. Therefore, it can be said that the validity and reliability reported for the paper version of questionnaire will also apply to the electronic version.

It is expected that the use of this application can improve the measured variables in the intervention group compared to the control group. Specifically, the research hypotheses are that patients in the intervention group have significant differences with those in the control group in terms of indicators of physical activity, depression, quality of life, medication adherence, satisfaction with treatment, and the number of complications.

##### Stroke severity and motor function

The (NIHSS) questionnaire will be used to measure stroke severity and motor function of upper and lower limbs. Two items (out of 11 items) of this questionnaire will be related to motor function of limbs [30]. The data obtained from this questionnaire will cover the section of body structure related to ICF. This questionnaire will have 11 items, each of which with 5 options and scores from 0 to 4 (a score of 0 indicates normal performance and a score of 4 indicates a severe defect in performance). Due to the special type of scoring, scores will eventually be obtained between 0 and 42, which will be divided as follows:

- 0: No stroke symptoms
- 1–4: Mild stroke
- 5–15: Medium stroke
- 16–20: Moderate to severe stroke
- 21–42: Severe stroke

The validity of this questionnaire was confirmed using clinical predictor validity and its reliability was approved with Cronbach's alpha of 0.95 in Hinkle's study [31]. It should be noted that, in the present study, the original version of this questionnaire will be used by a neurologist.

##### Activities of daily living

The Barthel Index (BI) questionnaire will be used to measure the activities of daily living (covering the activities section of ICF). This questionnaire includes 10 key activities of daily living including: feeding, bathing, grooming, dressing, bowels, bladder, toilet use, transfers (bed to chair and back), mobility (on level surfaces) and stairs. In each item, scores are: 0 (dependent), 5 (need for major help), 10 (need for minor help) and 15 (independent). Finally, the ranking of people is done as follows:

- 80–100: independent.
- 60–79: slightly dependent.
- 40–59: relatively dependent.
- 20–39: very dependent
- Less than 20: completely dependent [32].

The validity and reliability of the Persian version of this tool were confirmed in a study conducted by Tagharrobi et al. [33] The validity of the questionnaire was confirmed using concurrent validity, comparison of known groups and exploratory factor analysis and the reliability was confirmed using Cronbach's alpha. The Cronbach's alpha coefficient of this instrument was reported as 0.99.

##### Quality of life

To assess the quality of life and the level of social participation (covering the social participation section of the ICF), the EQ-5D-3L (EuroQol group-5 Dimentions-3 Level) questionnaire including 5 questions on a 3-point Likert scale will be used. The final score of the questionnaire will be between 0 and 10. The validity and reliability of the Persian version were confirmed in a study done by Dastourani et al. [34] The construct validity and the differential validity were confirmed by testing the predetermined hypotheses and by the method of known groups, respectively. Cronbach's alpha coefficient of this questionnaire was reported as 0.89.

##### Depression

To assess the depression (covering the body structure section of ICF), the BDI-II (Beck Depression Inventory-II) questionnaire will be used, which includes 21 groups of sentences with a value scale of 0 to 3 expressing the patient’s feelings during the previous 2 weeks. The scores of this questionnaire are between 0 and 63. The validity and reliability of the Persian version of this questionnaire were confirmed in a study done by Hamidi et al. [35] The validity of the questionnaire was confirmed using descriptive and confirmatory factor analysis as well as convergent validity and its Cronbach's alpha was 0.93.

##### Medication adherence

The MMAS-8 (Morisky Medication Adherence Scale-8) questionnaire will be used to assess patients' medication adherence. This questionnaire consists of 8 questions, of which items 1 to 7 are yes/no questions, and item 8 has a 5-point Likert scale. For items 1 to 7, for each no answer, a score of 1 and for each yes answer, a score of 0 will be recorded. For question 8, scores from 0 to 4 (never, rarely, sometimes, often, always) will be considered. The final score will be between 0 and 8, with 8 indicating high adherence, 6 and 7 indicating moderate adherence, and less than 6 indicating low adherence. The validity and reliability of the Persian version of this questionnaire were confirmed in a study conducted by Ghanei Gheshlagh et al. [36] The validity was confirmed using the concurrent criterion validity method and reliability. Cronbach's alpha coefficient was reported as 0.72 in this study.

##### Patient satisfaction

The PSQ (Patient Satisfaction Questionnaire) will be used to measure patient satisfaction with the treatment. This questionnaire includes 11 items with a 5-point Likert scale (from 0 for very poor to 4 for excellent). The total score is a number between 0 and 44. The scores of 0–10 mean poor satisfaction, scores 11–21 mean good satisfaction, scores 22–32 mean very good satisfaction and scores 33–44 mean excellent satisfaction. The validity and reliability of the Persian version of this questionnaire were confirmed in a study done by Yousefi Golafshani et al. [37] The face validity and content validity were confirmed using the opinions of 10 experts in the field of medical education and Cronbach's alpha was reported as 0.98.

##### Complications

To assess the frequency and type of complications in patients, a stroke complication checklist will be used which includes a total of 19 complications, eight of which are based on the most common stroke complications from the perspective of the American Heart Association (AHA) and the American Stroke Association (ASA) [38] and 11 of which are based on the most common complications reported in related studies [39–42]. This checklist will be prepared by the researchers and the face and content validity of this checklist will be confirmed by 3 neurologists.

#### Data analysis

Data analysis will be performed using IBM SPSS 22 [43]. The data will be entered anonymously using codes and numbers, which can be decoded only by one of the researchers (except the assessor or the analyst). The data will be stored in this software application and supported by a backup on another system kept in another place. The data will be first presented in the form of descriptive statistics and frequency distribution tables. The normality of the data will be checked using the Kolmogorov–Smirnov test. Depending on the type of data distribution, the mean or median of the data will be used as a criterion. In case of normal distribution of data, the mean will be used and in case of abnormal distribution (non-parametric), the median will be used for statistical calculations. In order to determine the relationship between the scores of patients’ activities of daily living, quality of life, depression, medication adherence and patient satisfaction with patients’ group type (control or intervention), an independent t-test will be used. If there is a significant difference between the intervention group and the control group, the effect of the intervention will be specified. Repeated measures analysis of variance (ANOVA) will also be used to analyze multivariate and control the effects of confounding variables on dependent variables. Confounding variables will include: patient age, sex and educational level, which can affect the final results. In all tests, a significance level of 0.05 will be considered. Pearson or Spearman correlation coefficient (according to the type of variable distribution) will be used to determine the relationship between the usage duration of the program and the score of the measured variables. After categorizing the time of application usage, one-way ANOVA or Kruskal–Wallis’s test (depending on the type of variable distribution) will be used to determine the relationship between the measured variables and the time of application usage. Chi-square test will be used to determine the relationship between the type of complications in both groups. Also, to determine the relationship between the scores of the measured variables before and after the intervention, a paired t-test will be used in each group separately. Hypothesis H0 will indicate the absence of correlation and Hypothesis H1 will indicate the existence of correlation between the measured variables.

## Discussion

Various protocols have been designed to evaluate the impact of mobile applications and trainings on the outcomes of patients with stroke [44–47]. However, these studies differ in terms of sample size, number of variables and outcomes to be measured, duration of intervention and protocol design. In Iran, a study in which first an application is designed using expert and patient needs assessments according to a standard guideline and then the impact of designed application on patients with stroke is evaluated has not been done yet. In other foreign studies, less attention has been paid to different aspects of ICF. However, in the present study, we will try to evaluate the outcomes based on valid and reliable questionnaires that are related to different sections of ICF.

In addition to providing educational materials via text and image, the application will provide appropriate exercises using educational video clips. It will also contain items such as reminders and warnings to take medications and doctor's appointments, the possibility of communicating with the therapist via chats, voice calls or video calls, the possibility of weekly goal setting, and writing notes related to the rate of goal achievement. It is expected that the use of this application will potentially improve and enhance the motor function of the upper and lower limbs, balance and walking, social participation and self-efficacy in the intervention group more than those of the control group. In similar studies [48–50], the positive effects of mobile applications on improving the motor function of limbs after stroke have been shown. However, some other studies [51, 52] have reported the use of this type of intervention on the motor function of the limbs without significantly improved effects. The reasons for this difference can be related to various factors such as the context of the society under study, the mobile application used and its capabilities, the duration of the intervention and the level of patient cooperation to use the application.

All instructions and exercises in the application will be approved by the experts to be considered safe for patients to minimize the possibility of causing harm. To eliminate the risk of possible harm to patients during exercise, they will be advised to start the training with an easy level and to make sure that the exercises are done in the presence of a caregiver, and that the way to receive support from a caregiver is fully taught in the application through videos. All of the ethical considerations in clinical trials (including Helsinki ethical codes) will be considered by researchers. If medical complications arise during the study; patients will be treated for free by the responsible physician. Compensation in this regard will be done according to the laws of the Medical System Organization.

For auditing the trial, after registering each sample, a researcher will monitor the patient registration process and assign them to either the control or intervention group using the admin-side application. The completion of questionnaires by patients will also be monitored by this person. If the questionnaires are not filled out, patients will be reminded though a phone call. Filling out the questionnaires will be audited two and three months after patient registration and the necessary actions will be done in case of possible delays. These actions will include sending reminders through the application, sending messages and making phone calls.

The most important limitation of this study is the possibility of lack of cooperation of some patients to participate in the study. Researchers will understand the specific conditions of patients with stroke and will fully explain the purposes of the study to them, but in any case, if patients, despite being informed about the goals and efforts of researchers to obtain their satisfaction, seem to be unwilling to participate in the study, according to the principle of informed and free consent, they will be excluded from the study at any time during the research. Another limitation is the lack of qualified patients at the study site, which will probably make the sampling longer, but will not harm the results of the study. However, larger trials with larger samples in centers with more patients are recommended. One of the operational problems in studies of this type is the possibility of patients not being able to work with smartphones. If the patient is not able to work with a smartphone, his/her caregiver will be consulted. Of course, this person needs to be someone such as a patient’s wife/husband or child who is always available to the patient.

It can be said that the most important strength of this trial is the development of an application based on the needs assessment of all stakeholders. This can be a great advantage over studies that use an off-the-shelf application because it will be designed and adapted to the needs of the population under study.

The ability to generalize the results of the study to similar populations should be somewhat cautious. Although the sample at of this study is based on the formula and data obtained from previous studies, it is nevertheless a relatively small sample. Therefore, the generalizability of the results of this study needs to be done with caution.

### Trial status

Expected recruitment start date is April 2022 (after the manuscript submission date) and the recruitment will be completed approximately in September 2022. Possible changes related to the protocol will be transferred to the Iranian Registry of Clinical Trials (IRCT) System and the necessary changes will be made and informed on that website.

## Data Availability

The datasets used and/or analyzed during the current study are available from the corresponding author on reasonable request.

## Abbreviations

AHA: American Hospital Association
ANOVA: Analysis of Variance
ASA: American Stroke Association
AU: Attribute of Usability
BDI: Beck Depression Index
BI: Barthel Index
CVI: Content Validity Index
CVR: Content Validity Ratio
CW: Cognitive Walkthrough
EQ-5D-3L: EuroQol group-5 Dimensions-3 Levels
ICF: International Classification of Functioning, disability and health
MMAS-8: Morisky Medication Adherence Scale-8 items
NIHSS: National Institute of Health Stroke Scale
